# Unveiling the process behind quality indicator selection in Portuguese primary healthcare - a qualitative study

**DOI:** 10.1186/s12875-025-03013-7

**Published:** 2025-11-25

**Authors:** Bianca Pires-Vieira, Julian Perelman, Mário Santos, Bruno Heleno

**Affiliations:** 1 Comprehensive Health Research Centre, Person-Centred Research Group, NOVA Medical School, Lisbon, Campo dos Mártires da Pátria 130, 1169-056 Portugal; 2 Comprehensive Health Research Centre, Efficiency, Incentives and Sustainability Group, LA Real, NOVA National School of Public Health, Lisbon, Portugal; 3 Comprehensive Health Research Centre, Methods and Data Analysis Group; NOVA National School of Public Health, Iscte - Instituto Universitário de Lisboa, CIES-Iscte, Lisbon, Portugal; 4 Comprehensive Health Research Centre, Person-Centered Research Group, LA-REAL, NOVA Medical School, NMS, Universidade NOVA de Lisboa, Lisboa, Portugal

**Keywords:** Primary health care, Quality indicators, health care, Health services research, Stakeholder participation, Pay-for-performance

## Abstract

**Background:**

Since 2008, Portugal’s primary healthcare (PHC) units have been evaluated and partially funded based on an extensive set of quality indicators. This study aimed to explore the process of quality indicator selection, and the criteria used to choose among indicators.

**Methods:**

We conducted a qualitative study from 2022 to 2023, combining documentary analysis of 38 meeting minutes and 19 appendices from the National Technical Committee (NTC) with semi-structured interviews of 10 NTC members responsible for selecting these indicators. Data were analysed using computer-assisted thematic analysis with an interpretive approach in MAXQDA, and findings were triangulated across sources. The study followed the Standards for Reporting Qualitative Research (SRQR) guideline.

**Results:**

Key findings highlighted the importance of indicator validity, emphasizing the need for indicators to accurately reflect clinical performance unaffected by available resources or patients’ characteristics. Relevance was also viewed as a major attribute, requiring alignment with National Health Service goals, the potential to reduce performance gaps, and scientific robustness. Feasibility (e.g. availability of data) and usability (capacity to enhance quality in practice) were also frequently mentioned. Participants raised concerns about their roles within the Committee, noting ambiguity between providing technical input and acting as political representatives, compounded by professional tensions. Many also reported a lack of clarity regarding how indicators were developed, prioritised, and supported by evidence, with limited transparency and communication throughout the process. This contrasts with best practices identified in the literature, which emphasize transparency, pilot testing, and clearly defined roles to ensure effective stakeholder engagement.

**Conclusions:**

Our findings contribute to the understanding of quality indicator selection in primary healthcare, highlighting the need for clear stakeholder roles, greater transparency, structured consensus-building, and inclusion of patient and public perspectives. Future research should be embedded within the indicator development process to support continuous learning and improvement.

**Supplementary Information:**

The online version contains supplementary material available at 10.1186/s12875-025-03013-7.

## Introduction

In Portugal, a major reform of primary health care started in 2006 and was fully implemented by 2008 [1, 2] One of the flagship measures was the creation of Family Health Units (FHU), characterised by small multidisciplinary teams, the assignment of a family physician to each patient, and a financing model including capitation and pay-for-performance (P4P) components, i.e. healthcare professionals facing individual and collective financial incentives tied to their clinical performance. This model was implemented on a voluntary basis, and each primary healthcare centre (re-branded as Personalized Health Care Units, PHCU) had the opportunity to apply for FHU status. The limited evidence has shown that the FHU model was associated, in the short term, with quality and efficiency gains [3] obtained not only because of P4P but also because of the model’s attractiveness, which incentivized PHCU to improve their outcomes to meet the standards for obtaining FHU status.

One of the key aspects of this reform is P4P. International literature shows that P4P increases the quantity and quality of outpatient care, but it is unclear whether it improves key clinical outcomes or enhances health equity [4, 5] P4P may contribute to overcoming the insufficient integration of care, while anecdotal evidence suggests it has reinforced the attractiveness of the general and family medicine specialty for physicians. P4P may also lead to negative unintended consequences, namely the exclusion of sicker patients [6, 7] neglect of non-incentivized services [8] or resentment and the worsening of care when financial incentives are withdrawn [9]. Therefore, to leverage the potential of P4P, healthcare systems need to find the right quality performance indicators and the right framework to measure the quality of infrastructure, workforce, processes, and ultimate outcomes.

Quality indicators are data routinely collected by healthcare systems. Individual quality indicators should be relevant to measure and record, both because there is compelling evidence that they relate to health outcomes and because there is either considerable variation in performance or relevant health disparities. Indicators should have scientifically acceptable measurement properties, namely validity and reliability. They should also be feasible, meaning that it is possible to collect data using existing or easily implemented processes, and that data is available in electronic sources. Quality indicators should be usable by different stakeholders (e.g. patients, providers, policymakers), ensuring accountability, transparency, and improvement actions [10].

While relevance, acceptable measurement properties, feasibility and usability are important attributes of a quality indicator, seldom does an indicator meet al.l criteria [11]. Stakeholders may need to make trade-offs between these criteria. For example, the incidence of diabetes microvascular complications (blindness, end-stage renal disease, amputation) may be among the most important data to collect to measure the adequacy of diabetes care, and data collection may be feasible using electronic health records. However, it does not have adequate measurement properties to compare the performance of different healthcare providers because they are rare outcomes, and differences among individual healthcare providers are due to patients’ observable or unobservable characteristics and not to actual differences in performance. Stakeholders may then choose to focus on process-related measures of care (e.g. fraction of patients undergoing retinal screening, microalbuminuria, or foot examination) which may have better measurement properties, but are less relevant than the actual complications, and may need healthcare provider cooperation to capture the elements of care. It is still unclear how stakeholders make these trade-offs.

In summary, the effectiveness of P4P depends on the choice of indicators, which is itself dependent on decision makers’ preferences regarding indicators’ attributes. In turn, these preferences are likely to be influenced by stakeholders’ knowledge and working context. Knowing that there are no “perfect” indicators that fulfil all relevant criteria, understanding the role of stakeholders’ preferences and their determinants is crucial to assess the indicators’ selection process and the functioning of P4P models.

In this article, we analyse the process of discussing and validating the indicators underpinning the P4P method in Portuguese primary health care, using a qualitative approach. To do so, we focused on the work of the National Technical Committee (NTC) - a group of key stakeholders who validate all indicators that directly influence the distribution of financial incentives to FHUs. We performed a documentary analysis of the NTC’s meeting minutes and conducted semi-structured interviews with members of the NTC.

## Methods

### Qualitative approach and research paradigm

We conducted a qualitative study from September 2022 through September 2023, using document analysis of meeting minutes and semi-structured interviews with members of the NTC. Data were analysed using a computer-assisted thematic analysis with an interpretive approach [12–14], in MaxQDA. This manuscript is structured in accordance with the Standards for Reporting Qualitative Research (SRQR) guideline [15].

### Context

In Portugal, the publicly funded National Health Service (NHS) provides comprehensive coverage to all citizens. The NHS coexists with social insurance systems covering some professional groups (e.g. civil servants), but around one third of health expenditures are financed by private sources (mostly co-payments to private providers, drugs, and exams) [16]. Primary healthcare (PHC) serves as the first point of contact with the public NHS, and family physicians act as gatekeepers to specialised care [17]. In primary care practices, preventive care, teams of family physicians, nurses, and administrative workers provide chronic illness management and acute illness care. Since 2006, Portugal’s public primary health care has evolved towards two main models: the Family Health Units (USF), and the Personalized Healthcare Units (UCSP). As of January 2022, approximately 72% of patients receiving public primary care were registered in USFs while 28% were registered in UCSPs [18]. 

The performance of all PHC settings has been measured through a list of indicators compiled into a Global Performance Indicator (IDG) [19]. One of the main differences between USFs and UCSPs is that USFs receive performance-based financial incentives related to the IDG value. Yet, to obtain the USF status, UCSPs also need to satisfy a list of quality indicators, meaning performance is evaluated in all PHC units, although for different purposes [1, 3]. Within public primary care, there is a single electronic health record system which allows healthcare professionals to access and manage patient health records digitally, including medical history, laboratory results, prescriptions, and appointment history [20, 21]. This data can also be used to monitor the performance of healthcare professionals and primary care practices.

The Central Administration of the Health System (ACSS) oversees the development of Healthcare Quality Indicators (QIs). In 2022 there were around 80 measurable indicators, organised in a framework with five principal dimensions: clinical performance, service provision, governance, training of healthcare professionals, and scientific output [19]. For instance, clinical performance encompasses aspects such as timeliness of care (e.g. same-day appointments), health promotion (e.g. proportion of pregnant women who got a first trimester ultrasound), disease management (e.g. proportion of people with diabetes aged 75 or younger who had an A1c > 8.0%), quality of prescriptions and orders (e.g. proportion of older people without a long-term prescription of benzodiazepines), and patient satisfaction (measured through patient satisfaction questionnaires). Governance includes indicators related to patient safety and continuous quality improvement (e.g. evidence of an antibiotic stewardship initiative). Service provision reflects the availability and use of services (e.g. number of clinician hours allocated to out-of-hours care), while training and scientific output capture involvement in education and research (e.g. identification of training needs).

QIs are automatically extracted from the electronic health record system. While the structure of the framework has remained stable since 2017, the set of indicators and relative weights of quality dimensions have evolved over time. Earlier versions (2007–2013) included fewer indicators with greater local flexibility; since then, the number and scope have expanded to include chronic disease management, potentially avoidable hospitalisations, and more granular measures of access and efficiency. Some indicators have been retired or replaced to reflect changing health priorities and data availability [22]. 

Proposals for specific QIs can originate from a variety of stakeholders, including the Health Ministry, the General Directorate for Health, or other stakeholders such as scientific medical societies. To address these proposals, the ACSS forms specialised task forces made up of external experts who guide the QI development process.

Following this, a NTC undertakes the discussion and validation of the QIs to be implemented in Pay-for-Performance schemes for primary healthcare teams. The NTC includes representatives of national (Ministry of Health, ACSS, General-Directorate of Health, National Coordination for Primary Care Reform) and regional agencies (five regional health administrations), professional bodies (Portuguese Medical Association, Portuguese Nursing Association, Family Health Unit Association, and Community Care Unit Association) and trade unions (two medical unions, three nursing unions, and no administrative staff unions). Patients’ or service users’ representatives are not included in the composition of the NTC.

The Committee also defines the acceptable range of variation in primary care team performance and identifies a narrower band representing optimal performance. Furthermore, it is within the NTC’s role to recommend new domains for healthcare quality evaluation and to suggest enhancements to existing quality indicators. According to its internal regulation, decisions are preferably made by unanimity but may be approved by a qualified two-thirds majority of the entities present, with each participating institution entitled to one vote. A quorum of at least nine entities is required for a valid decision.

### Sampling selection

The study included a documentary analysis of all minutes from the meetings of the NTC on the proposal, approval, or rejection of quality indicators. These meeting minutes were publicly available documents downloaded from the ACSS website. They recorded deliberations and final decisions of the Committee, and listed the names of the participants in each meeting. Documentary analysis was a useful first-stage method for systematically examining, the content of the NTC as well as their broader context – including the timeframe and gaps between meetings, the number and the professional identity of participants, and their expressed positions on the topics discussed.

The number of attendees and represented institutions varied between meetings. Minutes were taken by the ACSS. The first six meetings occurred bi-monthly; subsequent meetings were held at irregular intervals, ranging from monthly to annual, until January 2022. The minutes did not attribute statements or votes to specific individuals—only to the institutions they represented. While they provided an indirect and filtered perspective of what happened in each meeting, these documents were the best alternative to direct observation, to which we were not granted access, despite our endeavours. By analysing the minutes, we could grasp some of the dynamics between participating organisations and how these influenced the decisions, allowing for a first portrait of these meetings, which served as a guide for the preparation and conduction of the subsequent interviews. All 38 min were analysed from inception, in September 2017, to January 2022.

For the semi-structured interviews, we selected participants attending the meetings of the NTC, as they were involved in the decision making process regarding the quality indicators and, therefore, could offer key insights as to why some were either rejected or approved, and to the process behind the creation and development of the quality indicators. We included participants from different organisations (health authorities, unions, practising healthcare professionals, universities) and from different professional backgrounds (managers, nurses, and physicians). We started by interviewing an individual who had been involved in the meetings from the start. Afterwards, we used a snowball sampling method, asking each interviewee to recommend others who could contribute to the study. The final number of interviewees was determined iteratively. We initially set a target based on the scope of our research and the diversity of stakeholder perspectives we aimed to capture. As interviews progressed, we adjusted the number based on emerging findings and stopped recruitment when we reached thematic saturation — that is, when no new relevant insights were emerging from additional interviews.

### Data collection procedures

The data collected were of a qualitative nature. Data collection commenced in September 2022 and concluded in January 2023. An iterative process of data collection and analysis was employed, allowing for a deeper exploration of emerging themes. Starting with the documentary analysis allowed the research team to establish some initial ideas about which criteria were used by the NTC to select indicators. Based on an initial round of analysis of the minutes we designed the interview script for the semi-structured interviews. After 6 interviews, preliminary findings were discussed among the research team. This informed adjustments to the interview guide, both to improve clarity, and to further explore the process behind the discussion and validation of the quality indicators by the NTC. To ensure the robustness and validity of our findings, we triangulated our data sources. Insights from the documentary analysis were cross-referenced with themes emerging from the interviews. The combination of documentary analysis and semi-structured interviews allowed for a comprehensive understanding of both the institutional perspective and the opinions of stakeholders.

### Instruments and technologies to collect data

An interview guide was developed for this study (Supplementary File 1). It was divided into two main sections. After an initial set of questions to start the interview and make the participant feel more at ease with the interviewer, the first section focused on the interviewee’s personal description of the dynamics and events that took place in the meetings of the NTC, alongside any important aspects that were discussed there. This section aimed to provide a unique understanding of what was going on behind the scenes and how the decisions about the quality indicators were made, leading to rejection or approval by the members of the NTC. The second section focused on the type and content of discussions about the quality indicators and how they were evaluated in the NTC meetings. In this section, the interviewees were questioned about the criteria that were considered in relation to the quality indicators and how the process was developed during the meetings. They were also asked to reflect the desired characteristics of a quality indicator and, especially, criteria that should be considered when approving a quality indicator. After a preliminary discussion and analysis of the interviews, the research team added a section to the interview guide on the process behind the production of quality indicators before they were presented to the NTC, with the purpose of learning more about how NTC members perceived these first stages.

The interviews were all conducted by BPV, a health sociologist with experience in qualitative interviews. Participants could choose between a face-to-face or a remote interview. Most interviews were conducted remotely, through a video conferencing platform (ZOOM) [23]. The choice of the remote format was mostly for convenience and scheduling reasons. It also allowed us to interview participants from various geographical locations. Only one interviewee preferred a face-to-face interview. All remote interviews were video recorded, and the face-to-face interview was voice recorded.

Although we attempted to recruit a senior figure at the Central Administration of the Health System (ACSS) who had been directly involved in the development of quality indicators, our invitations received no response. At a later stage of the analysis, we met informally with other ACSS representatives to clarify specific aspects of the indicator development process prior to their presentation to the NTC. These interactions were intended for clarification purposes only and did not generate data included in the study.

### Description of the individuals involved in the study

Thirty-eight minutes from the NTC, and nineteen supplementary appendices were analysed. Twelve people were invited, and ten participants accepted the invitation to be interviewed. One union representative declined due to lack of agenda and suggested another participant from the same organisation. One researcher accepted, but did not reply to several attempts to schedule an interview. As mentioned above, one official at ACSS did not reply to the invitation.

The interviewees had all been practising their profession for more than ten years (see participants’ characteristics in Table 1). Six currently worked in a primary care practice, while others stated their main employer was a private clinic, a higher education institution or the health administration. Four were nurses, two of whom were currently working as academics. Five were doctors, one of whom was working as an academic. These diverse backgrounds contributed to different opinions and perceptions about the matters at hand.

**Table 1:** SOCIODEMOGRAPHIC CHARACTERISTICS OF THE PARTICIPANTS

Role (ID)	Gender	Years of professional experience	Main employer (*n*)
Primary care physicians (01, 02, 03, 07)	2 women, 2 men	15–34 years	Public health centre (3), higher education institution (1)
Primary care nurses (04, 05, 06, 08, 09)	2 women, 3 men	16–34 years	Public health centre (2), higher education institution (3)
High-level policy official (10)	1 man	20 years	Ministry of Health (1)

### Data processing

Two researchers collected and reviewed all documentary data (BPV and MS). Furthermore, after the interviews were conducted and recorded, all interviews were transcribed verbatim, by a single researcher (BPV), without the use of any transcribing software. Significant non-verbal cues, like prolonged pauses or evident emotional reactions, were noted within the transcripts. There was no cross-checking of interviews by a second interviewer. To protect participants’ identities, any identifying details (e.g. name, specific job title) were removed prior to transcription to ensure confidentiality. Interviews were conducted in Portuguese, transcribed in the original language, analysed, and then relevant excerpts were translated into English for the purpose of this manuscript. Initial translations of interview excerpts were performed by BPV, a native Portuguese speaker and fluent English user. These translations were then reviewed by BH to ensure fidelity to the original meaning. To enhance clarity and readability, we used a large language model (ChatGPT-4, OpenAI) in April 2024 to support final phrasing. The final version of each quote was cross-checked against the original by the research team to preserve accuracy and tone.

All interview recordings and transcriptions were stored on a secure, password-protected server. Only the research team had access to the raw data. Electronic data, including transcriptions and recordings, were stored on encrypted devices. Hard copies of notes and consent forms were kept in a locked cabinet in the primary researcher’s office. Plans are in place to destroy the recordings after 3 years, while transcriptions, with identifiers removed, will be retained for potential future research. After transcription, data were entered into MAXQDA software. Once all interviews were completed, a comprehensive analysis of both the documentary data and interview transcripts was conducted.

### Data analysis

Computer-assisted thematic analysis (CAT) refers to the use of software to support the identification and organization of patterns in qualitative data [13]. In our study, we used MAXQDA to facilitate the coding of transcripts, allowing us to systematically label excerpts according to a predefined and evolving list of codes. The software supported the management of a large volume of material, enabled easy retrieval and comparison of coded segments, and allowed us to iteratively refine themes throughout the analysis. While interpretation remained a human-driven process, computer assistance enhanced traditional thematic analysis by improving transparency, consistency, and efficiency in data organization and team collaboration.

Two researchers (BPV, MS) independently undertook the initial phase of inductive coding after reviewing the collected documentary data. This approach allowed for emergent themes and codes to be identified from the data, rather than from preconceived categories. Regular team meetings were held to discuss the evolving themes, helping calibrate their application and ensuring intercoder reliability. These discussions among team members, with different backgrounds and research experiences, promoted a shared understanding, refined the analytical framework, and contributed to analytical rigor.

Throughout the analysis, we maintained a reflexive stance—acknowledging and examining our assumptions, values, and potential biases—to ensure our interpretations remained grounded in the data. Interview transcripts were read in full, and relevant excerpts were coded. Once coding was complete across all interviews, we combined themes emerging from minutes and from the interviewees’ narratives. By cross-referencing these data sources, we ensured a deeper and more holistic understanding of the themes, confirming their presence across multiple data points and enhancing the validity of our findings.

In the later stages of analysis, we integrated established frameworks to further refine our insights. Specifically, we employed the National Quality Forum’s Measure Evaluation criteria for quality indicators (Importance, Scientific Acceptability of Measure Properties, Feasibility, Usability and Use). These frameworks provided a structured lens through which our data could be examined. The emerging results were continually discussed in a group setting and were edited with each meeting, as we were able to reach new conclusions, and as new ideas were brought up.

## Results

Table 1 presents the sample characteristics of the ten participants in the study. Four participated in more than ten meetings, as they had been members since the NTC was created. One participated in six to ten meetings, three participated in one to five meetings and one did not participate in any of the meetings. Table 2 describes the themes and subthemes that emerged from the data analysis.

**Table 2:** THEMES AND SUBTHEMES THAT EMERGED FROM THE DATA ANALYSIS

Themes	Sub-themes
Mandate of the NTC	Unclear mandate
	Adherence to the agenda
	Key moments of debate
Group dynamics within the NTC	Tensions between professional groups
	Perceived voting power
	Unclear process
Criteria to choose among indicators	Process of indicator evaluation and selection
	Relevance
	Measurement properties
	Feasibility
	Usability
Improving the decision making process	Vision for an ideal system of indicators
	Challenges in indicator development

*NTC: National Technical Committee*

### Actors and processes before NTC review

Our informants indicated the quality indicators were designed in response to public health challenges identified by the ACSS. The General Health Directorate, scientific societies, and professional organisations could also present target areas for quality indicator development. However, no formal process exists for suggesting indicators or areas, nor a predefined list of entities that can submit such proposals. To facilitate the development of these indicators, specialised working groups comprising experts from the relevant fields were formed. Afterwards, a process of evaluation was undertaken to assess the potential impact of each indicator and the feasibility of its implementation.

The operational dynamics within these groups were unclear, particularly regarding leadership and decision-making processes. It was not apparent how much of this preparatory work was communicated to the NTC members, as some expressed confusion about these foundational aspects during their interviews.

### Mandate uncertainty and group dynamics

A sizeable portion of the NTC meetings’ time was dedicated to clarifying its mandate and the scope of issues warranting its attention. These discussions often overshadowed those related to quality indicators. As shown in the following excerpt, the NTC spent considerable time debating what aspects it was free to discuss, instead of concentrating on the agenda items concerning quality indicators.

*“There were moments when it was discussed*,* which… what*,* for example*,* the powers of the National Technical Committee would be*,* whether we could be discussing certain issues…” (Interview 3)*.

The analysis revealed tangible professional tensions during discussions. Both doctors and nurses both contested each other’s legitimacy of the other group regarding the approval of quality indicators specifically related to their respective professions, casting a shadow of potential bias and legitimacy over the selection process. This is illustrated by the following excerpt:

*“Many times… the entities representing doctors felt that they did not have great competence to vote on an issue that was specific to the area of nursing and vice versa*,* right? Sometimes there were indicators more from the… medical field*,* right? Then there was the discussion about whether nurses should address these issues and vote in these more specific areas?” (Interview 3)*.

Furthermore, concerns were raised regarding perceived disparities in voting power among different professional groups. There was a feeling among nurse participants that physicians, being more numerous in representation, not only wielded greater voting power but also unduly influenced the decision-making process, especially regarding the endorsement of quality indicators. An interviewee reflected on this:

*“(…) I would say that our system is very medical-centred and therefore*,* there is an overvaluation of medical indicators that end up having a greater influence due to the lobby that is created*,* right? And therefore*,* I would say that in the Committee discussions it seems that medical indicators are valued differently compared to other indicators”* (Interview 6).

Some members also felt the process of creating quality indicators was unclear. Participants considered they were brought in to validate indicators suggested by others, and not to take an active role in their development. They noted a lack of information on how priority areas were defined, how indicators were selected, and what evidence supported the indicators. Interviewees mentioned that:

“*[About the criteria and dimensions] I don’t know*,* it was never very transparent… (…) in our case… it was to approve or disapprove the indicators. The person who constructed them (…) presented them to be appraised. (…) for some*,* we managed to correct [them]*,* taking care to ensure they were evidence-based…” (Interview 7)*.

*“. no… from a scientific point of view. I think there is no doubt about the importance of the indicator. The way it [the quality indicator] is evaluated and weighed… and that’s where it seems to me to be too random and confusing*,* therefore*,* too subjective.” (Interview 4)*.

This comments make it clear that the interviewees felt they were limited to voting on quality indicators and that the process was never made clear or transparent to them.

### Criteria to choose among indicators

The importance of an indicator lies in its ability to provide meaningful information that stakeholders will find useful for a specific purpose, such as improving health outcomes. Participants described various considerations that influenced the evaluation and selection of quality indicators. From their narratives, we identified four recurring ideas— relevance, measurement properties, feasibility, and usability —which appear as subthemes within the broader theme ‘Criteria to choose among indicators’. These ideas align with what is referred to in the quality indicator literature as ‘measure evaluation criteria’.

#### Relevance

The relevance refers to whether the specific measure focus is evidence-based, important to achieving significant gains in healthcare quality, and capable of improving health outcomes for a specific high-priority (high-impact) area where performance is variable or generally suboptimal. Four participants referred to this as a key consideration. A quality indicator helps improve performance in its own area and goes on to affect the entire system of indicators may be seen as beneficial, explaining why the interviewees were so concerned with relevance. One of them mentioned this broader perspective:

*“. this is a very important criterion. And how can that indicator*,* if improved*,* improve all the others? Because we still have very isolated indicators and then we cannot achieve results across the entire system…” (Interview 6)*.

Participants described five key considerations related to relevance. The first was goal alignment, meaning that quality indicators need to be aligned with the mission of the National Health Service and align with the previously established priorities. For example, this includes ensuring access to care and supporting the health of young children during their early years. This sub-criterion was also mentioned by two interviewees, namely:

“. *what are we committed to doing in a health service? Being on time*,* keeping an appointment schedule*,* having several schedule availabilities so that patients can access health services. After all*,* we are very important in the first years of life and in ensuring the functioning of the whole… the entire structure of the child and youth health service*,* because it will then have an impact throughout life. So*,* this has to do with what we commit to do*,* with the quality of what we commit to do (Interview 2)*.

Performance gaps refer to whether an indicator can highlight areas with potential for improvement. For example, if there is a very high level of unnecessary emergencies, an indicator that brings this to light can help identify existing performance gaps and guide solutions before they cause severe problems for the National Health Service. This was highlighted by three interviewees:

*“(…) How does this indicator enable us to improve? Because the indicator allows us to say “oh*,* there really are very few appointments!.” Oh*,* yes… “And why are there so few appointments?” [then] we were able to identify the [underlying] factors…*” *(Interview 6)*.

Scientific robustness refers to whether there is a reliable evidence base that demonstrates that the indicator is associated with better health outcomes. This was mentioned by five interviewees, making it the most frequently cited considerations related to relevance. Two representative quotes follow:

*“That was a lot harder to find. There were very few indicators where this evidence existed. So*,* what was chosen to do was a consensus among the experts*,* taking into account the results [that had been previously] obtained by the units.” (Interview 3)*.

*“. the indicators that I think have some strong evidence to support are those that have to do with diabetes and hypertension*,* and perhaps chronic obstructive pulmonary disease*,* but they are always subdued*,* because what is measured by diagnostic tests*,* and then vaccination*,* vaccination coverage rates*,* everything.” (Interview 1)*.

When evidence was scarce or unclear, interviewees often mentioned using previous indicator results, as a basis for judging whether practice variation was unacceptable.

*“. and it was in the absence of more robust scientific evidence… in the absence of more robust scientific decision*,* our proposal was grounded on the history of previous indicator results.“*.” *(Interview 3)*.

*“. the votes [against the indicator] … were based on the lack of information about previous indicator results*,* preventing the analysis of the implications…” (CTN Minute 31)*.

For the indicator to be relevant, some interviewees argued that indicators should reflect population preferences. As mentioned by one of the interviewees:

*“. and finally*,* there is another type of dimension that I think sometimes also brings some bias and here already brings some public participation. In other words*,* since it is also the opinion of communities and populations… fundamentally present here*,* when we have our electoral campaigns*,* whether at central level*,* regional level or local level*,* in which the population often values a lot more*,* for example*,* the creation of a cutting-edge service in a local hospital than it values*,* for example*,* the creation of a community intervention team in the same location…” (Interview 5)*.

The final aspect of relevance mentioned was content coverage, referring to the need for indicators to capture the full range of health quality dimensions. This means that quality indicators should cover a broad range of health domains and be applicable across as many healthcare units as possible.

*“The problem with having few indicators is that… we in primary health care… have a very large area of intervention*,* don’t we? In other words*,* more and more pathologies*,* more and more interventions.” (…) “That’s why initially there were only around 20 indicators and now we have 80. Because it was understood… for example*,* that mental health*,* osteoarticular disease or respiratory disease were also important and should also have some indicators.” (Interview 3)*.

*“If there are too many [indicators]*,* we are clearly lost.” (Interview 5)*.

Content coverage was not consensual among NTC members. Some felt that indicators should reflect full range of conditions managed in primary care, while others welcomed a smaller and more manageable set of indicators that clearly highlighted priorities in healthcare improvement.

#### Measurement properties

Measurement properties refer to whether an indicator is consistent (reliable) and credible (valid).

The reliability of the indicator refers to whether the measure is well defined and precisely specified so that it can be implemented consistently within and across organisations, allowing for comparability. To be trusted quality indicators need to be clearly defined, including the details about numerators and denominators. For example, the avoidable hospitalisation rates indicator should have a clear and uniform definition of what is “avoidable” and what is not, to prevent differential measurements in different settings. As mentioned by one of the interviewees:

*“this is […] something that those who work with indicators call the reliability of that indicator. [It is needed] If people are really going to trust it to evaluate that specific thing that it is evaluating… and sometimes that [reliability] does not exist.“” (Interview 6)*.

Before being adopted for quality measurement or pay-for-performance, new indicators underwent a period of testing where they were collected at all primary care units to check how stable results were and how they were correlated with other similar measures.

Validity refers to the indicator’s ability to capture what it is intended to measure, namely the most important facets of care.

*“. And if it is done well*,* then the indicators at the end serve to measure and for us to understand if we are getting closer to this objective or if we are moving away from this objective. If we wanted to*,* even if we did it well*,* naturally… the indicators would improve and demonstrate this improvement in quality*,* because we weren’t working with indicators*,* we were working with health needs and solving people’s needs…” (Interview 2)*.

*“. in fact*,* there are several dimensions that are taken into account when we talk about indicators and one of them has to do with the validity of the indicator itself*,* right? And how does this indicator contribute or not to the context you are evaluating? And*,* in fact*,* I would say that this criterion is the one that is least considered when we talk about indicators.” (Interview 6)*.

Capturing variation in clinical performance was, by far, the most frequently referenced aspect of validity—both by interviewees and in the NTC meeting minutes, where it was coded sixteen times. Two of the interviewees mentioned that:

*“. the service must meet certain criteria in terms of human resources*,* in terms of equipment*,* in terms of space*,* in terms of having certain characteristics*,* without which this training suitability is withdrawn by the Medical Association. And this also has an impact later on the organisation of the institution itself*,* for example*,* in the hospital or even in a primary care trust…” (Interview 5)*.

*“. as it considers that the indicator is not sensitive to the activities carried out in the units*,* taking into account the denominator of this indicator and the number of nurses allocated to these units*,* that is*,* more nurses would be needed to be able to develop specific and sufficiently robust work*,* capable of being able to influence this indicator.” (CTN Minute 36)*.

These quotations illustrate concerns about whether certain quality indicators are measuring the availability of human or technical resources or the clinical performance itself.

#### Feasibility

Feasibility refers to the extent to which the data required for performance measurement are readily available and processed without excess burden. Participants mentioned that the choice of quality indicators was often restricted to the data currently collected in electronic records. Some organisations suggested indicators that captured different dimensions of quality care (e.g. patient-centeredness), but such data were not routinely collected, or there were no known instruments that could reliably measure them.

*“There were some indicators that*,* despite being interesting and that everyone would agree to be included*,* ended up not being included due to IT issues and because the IT system was not prepared to collect this data.” (Interview 3)*.

Participants noted that indicators must adapt to the existing IT capacity; without this, it is not possible to record the information needed for performance measurement. This was pointed out by two of the interviewees and found in one of the minutes of the NTC commission.

#### Usability

This last section discusses the degree to which potential audiences such as patients, purchasers, providers, and policymakers are using or could use performance outcomes for the dual purposes of accountability and enhancing performance, with the aim of attaining high-quality, efficient healthcare. One participant noted:

*“An indicator must be useful for those who manage and those who finance [healthcare]… it must be useful for them*,* it must be objective and clearly measurable” (Interview 3)*.

Both the meeting minutes and participants mentioned that one aspect of the usability of quality indicators is helping to achieve efficient healthcare for individuals and populations. Efficiency was seen as the appropriate use of resources, at the lowest possible cost, avoiding waste. It was mentioned one time during the interviews, as we can see in the following excerpt:

*“I wish to build a system that not only enhances the efficiency of care management*,* but also ensures greater sustainability in financial investment. This system should be capable of integrating and amalgamating the diverse expertise present among various professionals (…) when the law specifies that a particular medical procedure is exclusively reserved for a certain professional class*,* we are unmistakably stating that the skills and possible competencies of other professional classes will not be considered in this context. Does this have an impact? Yes*,* it has cost implications.” (Interview 5)*.

*“There were some criteria that were primarily about costs*,* including those I previously mentioned*,* related to the expenses*,* treatment of the disease*,* and its management.” (Interview 4)*.

These quotations reflect participants’ clear concern with making the best use of available resources—financial or human—with the goal of building a more efficient healthcare system. However, while participants discussed the importance of usability from the perspective of policymakers and service managers, there were no references in either the interviews or the meeting minutes to the use of quality indicators by patients, service users, or the general public. Their role as potential users was acknowledged in general terms, but no concrete mechanisms for patient involvement or public-facing use of these indicators were identified in our data.

## Discussion

### Summary of the main results

In this study, we sought to identify the essential attributes of quality indicators in primary healthcare, as perceived by professionals involved in their development and implementation. Our findings confirm the importance of relevance, strong measurement properties, feasibility, and usability, with particular emphasis on validity. Furthermore, our results show that although governing policies ensured the inclusion of a wide range of stakeholders, this inclusivity led to unintended consequences. Many participants reported uncertainty about their specific roles —whether they were expected to contribute to the creation or simply to the endorsement of quality indicators. This lack of clarity was compounded by limited information about the indicators’ preparation phase, including the definition of priority areas, the process of indicator selection, and the appraisal of the supporting scientific evidence. In addition, professional tensions, and some members’ perceived lack of expertise further complicated discussions.

### Attributes of good quality indicators

During the stages of quality indicator development and selection, those addressing significant burden of disease, areas of substandard care or variability, and aspects where there is evidence that improving care leads to better patient outcomes should be prioritised [24]. In addition, the indicators must have robust measurement properties, be feasible, and understandable to their intended audience [25]. They should also align with national priorities, support quality improvement, and be clearly attributable to care providers [26]. These aspects resonate with the US National Quality Forum’s measurement quality criteria (“importance”, “scientific acceptability of measurement properties”, “feasibility” and “usability and use”, respectively), and are represented in our own data. Among all these desirable criteria, our participants emphasised the importance of validity (an aspect of measurement properties) and relevance.

The literature on quality indicators discusses various forms of validity: criterion, predictive, content, and construct [24, 27]. Our participants main concern appeared to relate to construct validity, i.e. the extent to which a QI is consistent with a theoretical framework. They often expressed concern over whether measurements accurately reflected the healthcare team’s performance instead of contextual factors. This concern may be grounded in studies suggesting that patient demographics, patient socioeconomic factors, and resource availability can influence the results of healthcare teams on quality indicators [28]. It may also reflect the ongoing crisis among primary care professionals, with high levels of dissatisfaction and burnout, related to working and employment conditions. Such conditions may reinforce the perception of an adverse environment that constrains professionals’ capacity to deliver optimal care.

Participants emphasised that quality indicators should be grounded on evidence that specific actions could improve patient outcomes, echoing findings from previous studies [26, 27]. However, perceptions of what makes an indicator relevant were shaped by additional factors. Some participants stressed the need for aligning indicators with key policy frameworks, such as the National Health Plan. Others highlighted the need to address known performance gaps and even used historical data to identify unacceptable performance variation.

### Decision making process: the blurred role of stakeholders

The NTC’s diverse membership, including representatives from government, professions, and unions resulted in unclear roles and, at times, interprofessional tensions. Discussions often focused on the appropriateness of cross-professional voting on indicators and the perceived imbalance in voting power. Furthermore, some members felt their role was merely to endorse indicators rather than contribute to their creation, with others doubting if they had the necessary expertise for the discussion. This blurred role was highlighted first by the name of the NTC, called as “Technical”, while its members were selected as representatives of their organisations, and not based on their technical knowledge about quality indicators. In other terms, the NTC was designed to achieve consensus among various stakeholders from a more political perspective, but meetings were organised around discussing technical questions. Second, the meeting preparation process (i.e., the purely technical exercise) was unclear for participants, without transparent information about how indicators were previously selected, and which frameworks and predefined criteria were used. Participants lacked access to the technical details of the process—details that they, at times, believed were within their remit to scrutinise. These problems were compounded by changing representations from organisations at meetings.

This contrasts sharply with best practices identified in the literature, such as the UK’s NICE process, which emphasises transparency, pilot testing, and clearly defined roles from the outset to ensure effective stakeholder engagement [26, 29]. In the UK, this process follows a clear script from start to finish. Participants are thoroughly informed about each step taken to reach a decision, with specific tasks assigned to named individuals or groups. Internationally, the process involves rigorous field testing and the use of frameworks that explicitly detail each phase, including evaluation against predefined criteria [30]. Formal consensus-building processes, such as Delphi panels and the modified RAND/UCLA appropriateness method have been used in quality indicator development [31, 32] and offer the added benefit of reducing bias and dominance in discussions. Such methods could have helped address some of the professional tensions and perceived voting power imbalances described in our results. These measures ensure that quality indicators are thoroughly vetted prior to implementation, contributing to more meaningful and equitable healthcare improvements.

While the NTC composition aimed to be diverse, no patient or service user representatives were included, and our data did not reveal any mechanisms for public consultation or participation in indicator development. Although quality indicators are available to the public [18] and, in principle, could support transparency or informed choice, their actual use appears to be limited to internal performance management and financing decisions. Usability from the perspective of patients or the general public was not raised by participants, suggesting that patient and public engagement was not a priority. A recent survey by the Organisation for Economic Co-operation and Development (OECD) found that primary care users in Portugal reported some of the poorest experiences with regard to patient-centred care and quality [33]. This may be partly explained by the lack of user involvement in the indicator selection process, which currently excludes measures of patient experience.

### Strengths and limitations

The strengths of our study lie in its dual approach of using both documentary sources and interviews, enabling a comprehensive understanding of the complexities involved in quality indicator development. Additionally, the diverse backgrounds of our research team enhanced the data analysis, bringing varied perspectives to the interpretation of findings. Importantly, the research was conducted in 2022–2023, nearly 15 years after the primary healthcare reform and the introduction of performance indicators. We have thus likely evaluated a mature and consolidated process, rather than an early-stage initiative still undergoing frequent changes.

However, the study also has limitations. The open-ended nature of our interview guide, while allowing for a broad range of responses, may have led to a lack of depth in certain areas. In retrospect, a more structured approach with explicit questions on each criterion and dimension of quality indicators might have yielded more focused insights. Furthermore, our inability to interview people involved in the creation of the quality indicators and the reliance on a non-representative subsample of the NTC members may limit the transferability of our findings.

### Future research

The limitations outlined above suggest that future research should not only examine indicator selection retrospectively, but also be embedded in the process, ideally through an ethnographic approach. That is, research should monitor and evaluate the development and implementation of quality indicators in real time, both to generate knowledge and to support continuous improvement. It should also explore the views and perceptions of frontline health professionals who are directly involved in reporting, using and being evaluated by these indicators, boing beyond the institutional or stakeholder-level views captured in our study. Finally, future research should explore mechanisms to incorporate patient and public perspectives, which remain largely absent from current indicator selection processes but are essential for ensuring the relevance, legitimacy, and acceptability of quality assessment in healthcare.

## Conclusion

Our study examines the selection process of quality indicators for Portuguese primary care, and the stakeholders involved stressed the importance of validity and relevance. Unclear roles, limited transparency, and professional tensions were noted, which appeared to hinder productive discussions. The absence of public or patient involvement also represents a notable gap.

Based on these findings, we propose several recommendations that may strengthen indicator selection processes both in Portugal and other health systems. First, future research should be embedded within the indicator development and implementation process to support ongoing learning and adjustment, rather than being limited to retrospective assessments. Second, it is important to clarify the roles of stakeholders involved in indicator committees, ensuring alignment between their mandate and the expectations of their contributions. Third, the development of indicators should follow a transparent, structured process, with clear criteria and evidence appraisal methods made available to all participants. Fourth, formal consensus-building techniques may help mitigate power imbalances and improve group decision-making. Fifth, mechanisms to incorporate patient and public perspectives should be established, as their voices were entirely absent in our case study. Finally, the usability of indicators should be assessed more broadly, considering their potential use not only for performance management but also for public accountability and patient empowerment.

## Supplementary Information

Supplementary Material 1

## Data Availability

The interviews analysed in this article are not publicly available because the study participants did not give consent for their data to be shared publicly. However, anonymized interview transcripts are available from the corresponding author upon reasonable request.
