# Supplementary material for: Unveiling the process behind quality indicator selection in Portuguese primary healthcare - a qualitative study
Source: BMC Prim Care. 2025 Nov 25;26:378. doi: 10.1186/s12875-025-03013-7 (PMC12648884; doi:10.1186/s12875-025-03013-7)
Supplement: Supplementary file 1 — Supplementary Material 1 [file 12875_2025_3013_MOESM1_ESM.docx]

# Interview Guide:

## Block A - Initial Questions / Icebreaker:

1. Can you tell me what your profession is?
2. How long have you been practicing it?
3. What is your main place of work?
4. Before having the opportunity to participate in the meetings of the National Technical Committee, were you aware that they existed? At that point, what would you have pointed out as the objectives of said meetings?
5. How many Technical Committee meetings have you participated in?

## Block B - Description of the meetings' functioning and how they were organised:

**Objective**: To understand the interviewee’s perception of the functioning and composition of the National Technical Committee meetings.

**Main Question**:

- Can you describe the functioning of the National Technical Committee meetings?

**Possible probing questions** (may or may not be used):

- In your own words, could you describe what a National Technical Committee meeting consisted of?
- Were there any defined operational rules? If so, what were they? Do you think they were followed during the duration of the meetings?
- Would you say that many representatives of the entities attended the meetings? If so, could you provide me with an estimate of how many were present?
- Which professional roles do you think were most represented in the meetings? And which were least represented?
- In your opinion, did the meetings follow the agenda that was previously established? If not, what was added? What was left out?

## Block C - Description of the events and dynamics within the meeting itself:

**Objective**: To understand the events/dynamics within the meetings and the relationships between doctors, nurses and other professionals.

**Main Question**:

- How would you describe the typical order of events in a National Technical Committee meeting?

**Possible probing questions** (may or may not be used):

- Were there some entities that participated more in the meetings than others? If so, in your opinion, what were the reasons behind this?
- How would you describe the relationships/dynamics between different professional roles during the meetings?
- What were the moments of greatest discussion and debate about? Primary healthcare indicators? Others?
- What types of activities/tasks would you say were most prioritized in the meetings? And what types do you think were not discussed as much and should have been?

## Block D - Reflection on the indicator discussion process:

**Objective**: To try to understand the process behind the selection of indicators and whether or not they occupied a prominent place during discussions at the meetings.

**Main Question**:

- Keeping in mind the last meeting you attended, could you describe how the process of recording, selecting, and evaluating the indicators took place?

**Probing Questions**:

- For you, how would an ideal indicator system be designed? Would many or few indicators be chosen?
- What criteria do you think were considered in the decision-making process regarding the indicators, i.e., in their rejection or approval by the entities? In your opinion, which criteria do you think were missing and should be considered?
- What dimensions do you think were considered in the indicator selection process? In your opinion, which dimensions do you think were missing and should be considered?
- In your opinion, should the indicators be about process (e.g., proportion of patients with hypertension receiving proper follow-up) or outcome (e.g., avoidable hospitalization rate)?
- What role would you say the lack of IT resources plays in the selection of indicators? Do you think some indicators are left out/rejected because health units are unable to meet the requirements?
- What role do you think the structure should play in the selection of indicators?
- What difficulties and limitations do you think exist in the process of choosing, deciding, and evaluating indicators? In your opinion, how can these be overcome?
- Lastly, would you like to add anything to what has already been said?
